# Postoperative Findings Mimicking Duodenal Leak After Graham Patch Repair: Intact Repair With Retroperitoneal Abscess

**DOI:** 10.7759/cureus.108259

**Published:** 2026-05-04

**Authors:** Newton Rahming, Meghana Singh, Stephan Corcho, Taylor Tomko, Frederick Tiesenga

**Affiliations:** 1 Surgery, Caribbean Medical University, Willemstad, CUW; 2 Medicine and Surgery, Umm Al Quwain Hospital, Emirates Health Services, Umm Al Quwain, ARE; 3 General Surgery, Community First Medical Center, Chiacgo, USA; 4 Surgery, St George's University, St. George’s, GRD; 5 School of Medicine, American University of Barbados, Bridgetown, BRB; 6 General Surgery, West Suburban Medical Center, Chicago, USA

**Keywords:** controlled drainage, duodenal leak, duodenal ulcer, graham patch repair, postoperative complication, spontaneous closure

## Abstract

Perforated duodenal ulcers represent a surgical emergency requiring prompt operative intervention. The Graham omental patch is the most commonly performed procedure for emergent repair; however, postoperative findings suggestive of leakage remain a serious concern and are associated with significant morbidity and mortality. Traditionally, suspected postoperative leaks have prompted early surgical re-exploration due to the risk of peritonitis and sepsis. However, select patients with concerning postoperative findings may be managed successfully with appropriate drainage, decompression, and nutritional support. We present the case of a 79-year-old male patient who was brought to the emergency department with progressive altered mental status and generalized abdominal pain. Imaging revealed findings consistent with a perforated duodenal ulcer, and the patient underwent emergent exploratory laparotomy with Graham patch repair and placement of a Jackson-Pratt drain. During the postoperative period, bilious output from the drain raised concern for a possible duodenal leak. A contrast-enhanced CT scan with enteric (oral) contrast demonstrated contrast entering the drain, prompting surgical re-exploration. Intraoperatively, the previous repair was found to be intact without evidence of active leakage; instead, a retroperitoneal abscess extending along the duodenal sweep was identified and drained. With continued supportive management, including nasogastric decompression, antibiotics, proton-pump inhibitor therapy, and nutritional support via gastrostomy tube, the patient demonstrated progressive clinical improvement. Drain output gradually diminished and ceased, consistent with resolution of the postoperative process. This case highlights that postoperative findings suggestive of duodenal leak may not represent true failure of the primary repair and underscores the importance of correlating radiologic, intraoperative, and clinical findings. Careful assessment and appropriate supportive management may allow for favorable outcomes in selected hemodynamically stable patients following Graham patch repair.

## Introduction

Perforated duodenal ulcers represent a rare but potentially life-threatening surgical emergency that frequently requires urgent operative intervention. Although their overall incidence is relatively low in the general population, they account for a significant proportion of surgical emergencies and are associated with substantial morbidity and mortality [1]. Peptic ulcer disease remains one of the leading causes of duodenal perforation and is commonly associated with chronic nonsteroidal anti-inflammatory drug use and *Helicobacter pylori *infection [1,2]. These perforations may present as either free or contained. In free perforations, intestinal contents leak into the peritoneal cavity and result in diffuse peritonitis, necessitating emergent surgical management [1]. In contrast, contained perforations occur when adjacent structures, such as the pancreas or omentum, limit contamination and localize the defect. Clinically, patients with duodenal perforation often present with acute abdominal pain, signs of peritonitis [2], and systemic inflammatory response, requiring rapid diagnosis and intervention to prevent progression to sepsis and multiorgan failure.

Simple closure reinforced with an omental patch, commonly referred to as a Graham patch, remains the standard treatment in both emergent and resource-limited settings [3]. This technique involves primary closure of the duodenal perforation followed by placement of a segment of well-vascularized omentum over the defect, which is secured in place to reinforce the repair and promote sealing through local vascular supply and tissue adherence. The omental patch acts as a biologic scaffold, helping to contain leakage and support healing of the underlying duodenal wall. It has been widely adopted due to its technical simplicity and effectiveness in managing small perforations [4]. Despite its success, postoperative findings suggestive of duodenal leakage remain a significant concern for morbidity and mortality [5]. Reported rates of postoperative leakage range from approximately 4% to 15% in contemporary series [6,7].

Historically, suspicion of postoperative duodenal leak following Graham patch repair was considered an indication for immediate surgical re-exploration due to the highly caustic and contaminated nature of duodenal contents [2]. However, more recent evidence suggests that conservative management may be successful in selected patients whose leaks are contained or adequately controlled by surgical drainage [7,8]. Despite these advances, distinguishing true postoperative duodenal leak from other postoperative findings remains a significant clinical challenge, particularly when imaging and clinical findings are discordant.

This report describes the case of a patient with postoperative findings concerning for duodenal leak following Graham patch repair, in whom re-exploration demonstrated an intact repair and a retroperitoneal abscess rather than an active leak. The primary objective of this report is to highlight the diagnostic challenges associated with postoperative findings that may mimic duodenal leak. A secondary aim is to emphasize the importance of integrating radiologic, intraoperative, and clinical findings when evaluating suspected postoperative complications. By illustrating the potential for false-positive imaging in this setting, this case underscores the need for careful, individualized clinical decision-making.

## Case presentation

A 79-year-old male patient was brought to the emergency department by emergency medical services due to progressive altered mental status and functional decline over several days. According to emergency responders, the patient had recently transitioned from being independent in ambulation and self-care to being increasingly lethargic and unable to perform activities of daily living. Further information was later obtained from the patient’s son, who reported that the patient had been found lying on the floor for an unknown duration and may have experienced multiple recent falls. The exact duration of symptoms prior to presentation could not be definitively established; however, the history suggested a delay of at least 24-48 hours prior to presentation.

Upon arrival, the patient appeared frail, disheveled, and malnourished. Vital signs were stable, with a blood pressure of 130/64 mmHg, heart rate of 88 beats per minute, respiratory rate of 20 breaths per minute, temperature of 36.8°C, and oxygen saturation of 95% on room air. Physical examination revealed diffuse abdominal tenderness with guarding. The abdomen was soft but tender to palpation without rebound tenderness. Bilateral lower extremity edema and multiple ecchymoses were noted. Neurologically, the patient was alert but confused, consistent with his baseline dementia.

Laboratory studies demonstrated blood urea nitrogen of 31 mg/dL, aspartate aminotransferase of 92 IU/L, and total bilirubin of 1.5 mg/dL. Creatine kinase was markedly elevated at 2102 IU/L, consistent with rhabdomyolysis likely secondary to prolonged immobility. White blood cell count was 8.9 ×10⁹/L, and lactate was 1.8 mmol/L, indicating the absence of significant leukocytosis or tissue hypoperfusion despite intra-abdominal pathology. Serum albumin was 3.6 g/dL on admission and decreased to 3.2 g/dL during hospitalization, consistent with borderline hypoalbuminemia and possible malnutrition. Initial laboratory findings are summarized in Table 1.

**Table 1:** Laboratory findings at admission These findings highlight a discordance between significant intra-abdominal pathology and relatively non-severe inflammatory markers. BUN, blood urea nitrogen; AST, aspartate aminotransferase; ALT, alanine aminotransferase; CK, creatine kinase; WBC, white blood cell count.

Laboratory Test	Patient Value	Reference Range	Interpretation
White Blood Cell Count	8.9 ×10⁹/L	4.0–11.0 ×10⁹/L	Within normal limits
Hemoglobin	14.1 g/dL	13.0–17.0 g/dL	Normal
Hematocrit	40.3%	38.6–49.2%	Normal
Platelets	318 ×10⁹/L	150–450 ×10⁹/L	Normal
Blood Urea Nitrogen (BUN)	31 mg/dL	7–25 mg/dL	Elevated
Creatinine	0.83 mg/dL	0.6–1.3 mg/dL	Normal
Sodium	137 mmol/L	133–144 mmol/L	Normal
Potassium	3.9 mmol/L	3.5–5.1 mmol/L	Normal
Chloride	100 mmol/L	98–109 mmol/L	Normal
Aspartate aminotransferase (AST)	92 IU/L	13–39 IU/L	Elevated
Alanine aminotransferase (ALT)	37 IU/L	7–52 IU/L	Normal
Total Bilirubin	1.5 mg/dL	0.0–1.0 mg/dL	Mildly elevated
Albumin	3.6 g/dL	3.5–5.7 g/dL	Borderline low
Creatine Kinase (CK)	2102 IU/L	30–223 IU/L	Markedly elevated
Lactate	1.8 mmol/L	0.2–2.2 mmol/L	Normal
Lipase	7 U/L	11–82 U/L	Low
Glucose	110 mg/dL	70–99 mg/dL	Mildly elevated

**Figure 1:**
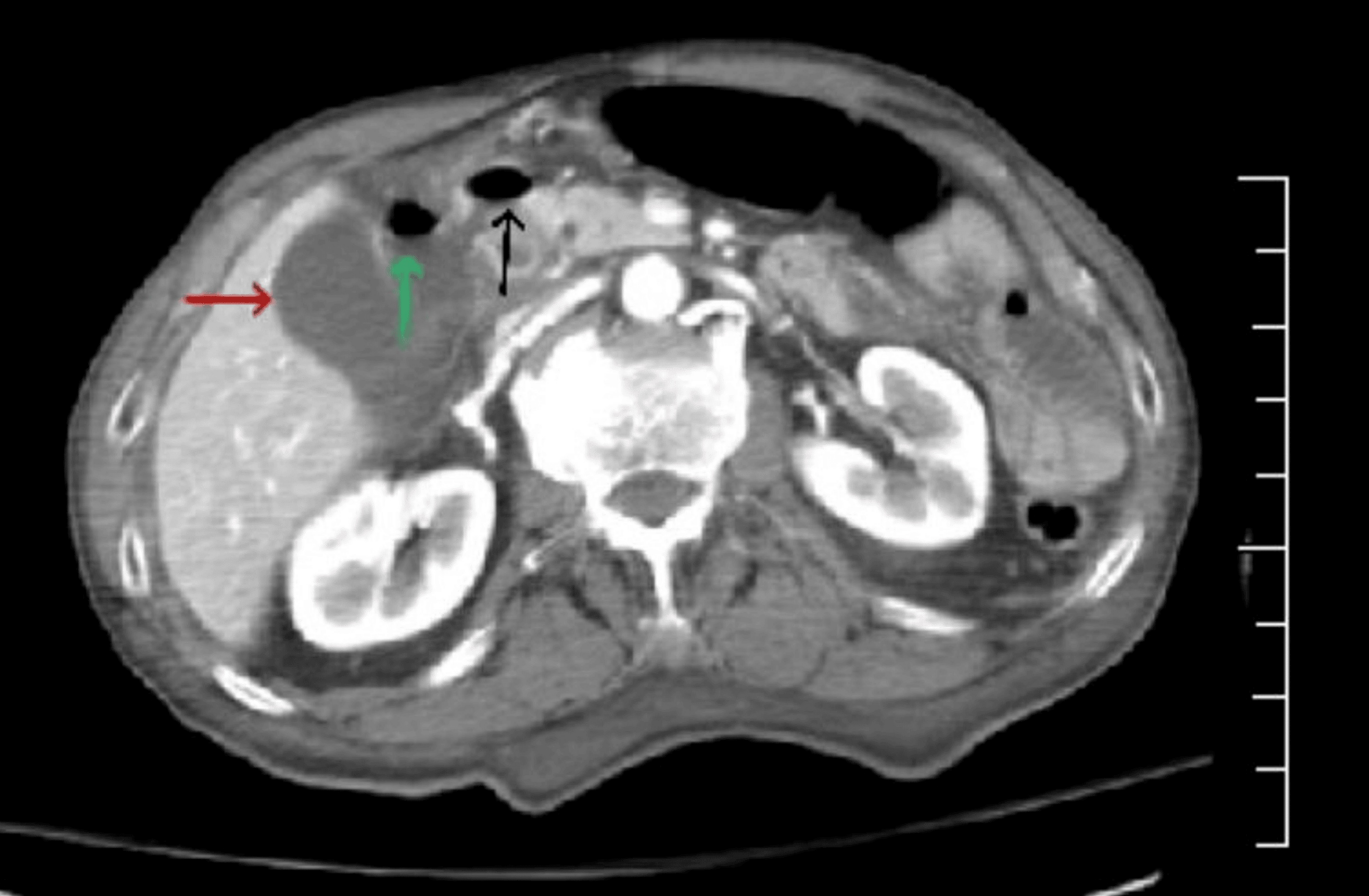
Contrast-enhanced axial CT of the abdomen demonstrating the gallbladder (red arrow) with adjacent extraluminal air (green and black arrows) and fluid in the subhepatic space between the gallbladder and duodenum These findings are highly suspicious for a perforated duodenal ulcer

Contrast-enhanced computed tomography (CT) of the abdomen and pelvis (Figure 1) demonstrated free intraperitoneal air with associated fluid in the subhepatic space between the gallbladder and duodenum, findings highly suspicious for a perforated duodenal ulcer. No alternative intra-abdominal source of perforation was identified.

Given these findings, the patient underwent emergent exploratory laparotomy. Upon entering the peritoneal cavity, turbid fluid and bile were encountered within the upper abdomen. Further exploration revealed a perforation in the first portion of the duodenum. The size of the perforation was not formally documented in the operative report. The defect was repaired using a Graham omental patch, and the abdomen was irrigated extensively. A Jackson-Pratt drain was placed adjacent to the repair site prior to closure.

The patient was transferred to the intensive care unit for postoperative monitoring and managed with intravenous fluids, broad-spectrum antibiotics, proton pump inhibitor therapy, and nasogastric decompression. On postoperative day three, bilious fluid was noted in the surgical drain. Over postoperative days 3-5, drain output increased as summarized in Table 2, raising concern for a possible postoperative duodenal leak. In this case, suspicion for a leak was based on a change in drain output in conjunction with imaging findings demonstrating contrast opacification of the drain.

**Table 2:** Postoperative clinical timeline and drain output This table summarizes the chronological progression of clinical events and drain output following surgical intervention, including postoperative findings concerning for leak, re-exploration findings, and subsequent recovery. POD, Postoperative day; CT, Computed tomography; LLQ, left lower quadrant; RLQ, Right lower quadrant.

Hospital Day/POD	Clinical Event/Drain Output
Day 1	Admission with altered mental status and abdominal pain
Day 1	CT imaging: perforated duodenal ulcer
Day 1	Exploratory laparotomy with Graham patch repair
POD 3	25 mL serosanguinous output; bilious drainage noted from Jackson-Pratt drain
POD 4	35mL serosanguinous output
POD 5	Imaging suspicious for postoperative duodenal leak
POD 6	Surgical evaluation for suspected leak; LLQ: 55 mL, RLQ: 45 mL serosanguinous
POD 6	Re-exploratory laparotomy; retroperitoneal abscess drainage and gastrostomy tube placement
POD 7	35 mL output from both right and left drains
POD 8	Right: 20 mL, Left: 30 mL
POD 10	Right: 20 mL light serous, Left: 10 mL serous; significant decrease in drain output
POD 14	Right: 10 mL light serous, Left: 5 mL serous; significant decrease in drain output
Discharge	Patient discharged in stable condition

A contrast-enhanced CT scan with enteric (oral) contrast demonstrated contrast entering the drain. However, such findings are not specific for luminal leakage and may reflect drain proximity, contrast tracking through inflamed tissues, or communication with a postoperative abscess cavity rather than direct communication with the duodenal lumen. These findings were therefore interpreted as concerning but not definitive for a leak.

Despite hemodynamic stability, re-exploration was pursued on postoperative day six due to increasing drain output, imaging findings suggestive of leak, and the inability to exclude a clinically significant duodenal leak that could progress to sepsis if untreated. During re-exploration, the previous Graham patch repair was found to be intact without evidence of active leakage. Further exploration revealed a partially intra-abdominal and partially retroperitoneal abscess extending along the duodenal sweep toward the ligament of Treitz. The abscess was drained, cultures were obtained, and an additional Jackson-Pratt drain was placed. Due to the patient’s significant malnutrition and poor oral intake, a gastrostomy tube was placed to facilitate enteral nutrition.

Over the subsequent 7-10 days, the patient’s condition gradually improved. Drain output progressively decreased, nasogastric output diminished, and clinical status stabilized as shown in Table 2. Enteral nutrition was initiated via the gastrostomy tube and left in place following discharge, with improvement in overall condition. The combination of improving clinical status and decreasing drain output suggested resolution of the postoperative collection.

The patient was discharged in stable condition. At follow-up, he remained clinically well, tolerating enteral nutrition without evidence of recurrent abdominal symptoms.

## Discussion

Duodenal perforation is a rare but potentially lethal condition, with reported mortality rates ranging from 4% to 30% depending on patient comorbidities, delay in presentation, and severity of contamination. Peptic ulcer disease remains one of the most common causes of duodenal perforation and is frequently associated with chronic nonsteroidal anti-inflammatory drug use and *H. pylori* infection. These perforations may present as either free or contained. In free perforations, intestinal contents leak into the peritoneal cavity and result in diffuse peritonitis requiring urgent surgical intervention [1]. In contrast, contained perforations may be localized by adjacent structures and occasionally managed conservatively.

Although the majority of surgical repairs are successful, postoperative leaks have been reported in approximately 4-15% of cases [6,7]. These leaks represent a serious complication and may lead to intra-abdominal sepsis, abscess formation, and increased mortality. Delayed presentation beyond 48 hours has been identified as a significant risk factor for repair failure. Larger perforation size and sepsis at presentation further increase the likelihood of postoperative leakage [8]. Poor nutritional status, particularly hypoalbuminemia, has also been associated with increased risk of leak and mortality [7]. Patients presenting in septic shock or with prolonged perforation duration are at especially high risk of postoperative complications [9]. Predictive models incorporating clinical variables have further emphasized the multifactorial nature of this complication [6]. In addition, certain etiologies of duodenal perforation, such as endoscopic retrograde cholangiopancreatography (ERCP)-related injury, have been associated with a higher likelihood of postoperative leak compared with ulcer-related perforations [6]. These findings underscore the importance of individualized risk assessment when evaluating patients for postoperative complications following duodenal ulcer repair.

Although generally reliable, the Graham patch is not immune to postoperative failure, with reported leak rates ranging from approximately 4% to 15% in contemporary series [6,7]. Perforated ulcers frequently involve chronically inflamed, ischemic, or friable tissue that may not hold sutures effectively even after debridement [6]. In addition, the omental flap must adhere securely to the defect for optimal sealing, but local edema, inflammation, or impaired vascularity may delay this process. Mechanical factors such as tension at the repair site can lead to micro-separation of the patch, particularly in elderly or malnourished patients [7]. Increased intraluminal pressure from retching or early enteral feeding may further stress the repair, while exposure to bile and pancreatic secretions may weaken surrounding tissues and promote erosion at the suture line. Technical factors, including inadequate debridement or insufficient omental coverage, may also predispose the repair to early breakdown [10]. Together, these factors may result in postoperative findings that raise concern for leak or localized complications.

In previously reported series, confirmed postoperative duodenal leaks are typically associated with clinical deterioration, including signs of sepsis, hemodynamic instability, and persistent high-output bilious drainage, often necessitating urgent re-intervention [6,7]. In contrast, the present case demonstrated a discordance between imaging findings and intraoperative results, as the patient remained hemodynamically stable despite imaging suggestive of a leak. This discrepancy highlights an important distinction between true repair failure and postoperative collections that may mimic a leak on imaging.

In the present case, postoperative imaging raised suspicion for a duodenal leak due to the presence of contrast within the surgical drain. However, re-exploration demonstrated an intact Graham patch repair and instead revealed a localized intra-abdominal and retroperitoneal abscess. This finding is consistent with prior reports demonstrating that postoperative abscesses and localized fluid collections may simulate leak on imaging, particularly in the early postoperative period when inflammation and tissue disruption are present [10,11]. Unlike cases of confirmed leak described in the literature, which are frequently associated with worsening clinical status, this patient’s stability allowed for a more measured and individualized approach to management.

Importantly, in the postoperative setting, contrast opacification of a surgical drain is not specific for duodenal leak. Such findings may result from drain proximity to the repair site, contrast tracking through inflamed or disrupted tissues, or communication with a postoperative abscess cavity rather than direct luminal leakage. In addition, the diagnostic accuracy of contrast-based imaging in this context is limited, and false-positive findings have been described [10,11]. 

A key consideration in this case is the distinction between suspected and confirmed postoperative pathology. While imaging findings raised concern for duodenal leak, these findings represented a diagnostic hypothesis rather than definitive evidence of repair failure. Intraoperative evaluation ultimately confirmed an intact Graham patch and identified a retroperitoneal abscess as the true source of the postoperative findings. This highlights an important diagnostic pitfall in the postoperative setting, where imaging abnormalities may be overinterpreted without adequate consideration of alternative explanations. The discrepancy observed in this case highlights the limitations of contrast-based imaging and underscores the importance of correlating radiologic findings with operative and clinical assessment.

The present case further demonstrates that imaging findings alone should not guide management decisions in the absence of clinical deterioration. In this scenario, surgical re-exploration was justified due to increasing bilious output and the inability to exclude a clinically significant leak, despite the patient’s hemodynamic stability.

Several operative and non-operative strategies have been described for managing postoperative duodenal leaks, including jejunal serosal patch repair, T-tube duodenostomy, subhepatic drainage with revision of the omental patch, triple-tube ostomy, and conservative management with controlled drainage. Omental patch repair has demonstrated favorable outcomes in selected patients [10]. Alternative operative strategies have shown variable success depending on patient condition and timing of intervention [11]. Overall mortality remains high in patients who develop true postoperative leaks, with septicemia representing the leading cause of death [10].

Sepsis and septic shock remain among the most common complications associated with intestinal perforation and may occur in up to 30% of cases [1]. Comparative studies have demonstrated similar outcomes between omental patch repair and alternative techniques such as falciform ligament patch repair, suggesting that both approaches may be effective when appropriately applied [12].

Postoperative management traditionally includes nasogastric decompression and delayed enteral nutrition. Enhanced recovery after surgery protocols advocate early enteral feeding, avoidance of prolonged nasogastric decompression, and early mobilization to improve recovery [13]. However, implementation of these protocols in emergency surgical settings remains challenging due to the urgency of presentation and clinical instability of many patients.

This case highlights the importance of correlating imaging findings with intraoperative and clinical assessment, as not all suspected postoperative leaks represent true failure of the primary repair. In this case, the presumed diagnosis of postoperative leak was not confirmed intraoperatively and is better interpreted as diagnostic uncertainty rather than a true leak.

As a single-patient case report, its findings may not be generalizable to patients with hemodynamic instability, diffuse peritonitis, or confirmed postoperative leaks requiring urgent intervention. However, it provides important clinical insight into the interpretation of postoperative imaging and supports careful, individualized decision-making in similar cases.

## Conclusions

This case suggests that postoperative findings concerning for duodenal leak, including bilious drain output and contrast opacification of surgical drains, may not necessarily indicate failure of the primary Graham patch repair. In this patient, re-exploration demonstrated an intact repair and a retroperitoneal abscess rather than a confirmed active leak, highlighting the possibility of false-positive imaging in the postoperative setting. These findings highlight the importance of correlating imaging results with clinical status and intraoperative findings before drawing conclusions about repair failure. Controlled drainage, decompression, antibiotics, and nutritional support may support resolution of postoperative collections in selected hemodynamically stable patients. 
